# Unrecognized Emergence of Chikungunya Virus during a Zika Virus Outbreak in Salvador, Brazil

**DOI:** 10.1371/journal.pntd.0005334

**Published:** 2017-01-23

**Authors:** Cristiane W. Cardoso, Mariana Kikuti, Ana Paula P. B. Prates, Igor A. D. Paploski, Laura B. Tauro, Monaise M. O. Silva, Perla Santana, Marta F. S. Rego, Mitermayer G. Reis, Uriel Kitron, Guilherme S. Ribeiro

**Affiliations:** 1 Secretaria Municipal de Saúde de Salvador, Salvador, Bahia, Brazil; 2 Centro de Pesquisas Gonçalo Moniz, Fundação Oswaldo Cruz, Salvador, Bahia, Brazil; 3 Instituto de Saúde Coletiva, Universidade Federal da Bahia, Salvador, Bahia, Brazil; 4 Laboratório Central de Saúde Pública, Secretaria de Saúde do Estado da Bahia, Salvador, Bahia, Brazil; 5 Faculdade de Medicina, Universidade Federal da Bahia, Salvador, Bahia, Brazil; 6 Department of Environmental Sciences, Emory University, Atlanta, Georgia, United States of America; Aix Marseille University, Institute of Research for Development, and EHESP School of Public Health, FRANCE

## Abstract

**Background:**

Chikungunya virus (CHIKV) entered Brazil in 2014, causing a large outbreak in Feira de Santana, state of Bahia. Although cases have been recorded in Salvador, the capital of Bahia, located ~100 km of Feira de Santana, CHIKV transmission has not been perceived to occur epidemically, largely contrasting with the Zika virus (ZIKV) outbreak and ensuing complications reaching the city in 2015.

**Methodology/Principal Findings:**

This study aimed to determine the intensity of CHIKV transmission in Salvador between November 2014 and April 2016. Results of all the CHIKV laboratory tests performed in the public sector were obtained and the frequency of positivity was analyzed by epidemiological week. Of the 2,736 tests analyzed, 456 (16.7%) were positive. An increasing in the positivity rate was observed, starting in January/2015, and peaking at 68% in August, shortly after the exanthematous illness outbreak attributed to ZIKV.

**Conclusions/Significance:**

Public health authorities and health professionals did not immediately detect the increase in CHIKV cases, likely because all the attention was directed to the ZIKV outbreak and ensuing complications. It is important that regions in the world that harbor arbovirus vectors and did not experience intense ZIKV and CHIKV transmission be prepared for the potential co-emergence of these two viruses.

## Author Summary

Since 2014, Brazil has experienced simultaneous transmission of dengue (DENV), chikungunya (CHIKV) and Zika virus (ZIKV), hampering clinical differentiation of infections due to the close manifestations caused by these viruses. Salvador, Brazil’s third largest city, was one of the cities most affected by those arboviral diseases. While a large acute exantematous illness (AEI) outbreak attributed to ZIKV has been recognized in the city, transmission of CHIKV appeared to have been much less intense. To obtain a comprehensible overview of the recent epidemiological situation, we investigated the proportion of chikungunya positive results among Salvador residents that were tested for CHIKV infections from November 2014 to April 2016. We demonstrated a high rate of CHIKV detection in serum samples during June-November 2015, shortly after an AEI outbreak attributed to ZIKV. The increase in CHIKV cases was not promptly detected by public health authorities and health professionals, likely because all the attention was directed to the ZIKV outbreak and its ensuing complications. It is important for regions that harbor *Ae*. *aegypti* (and potentially other vectors), but have not yet been subject to much transmission of ZIKV, CHIKV, DENV (and to potential other new and emerging arbovirus), to be prepared for the potential co-circulation of these viruses and associated diagnostic challenges.

## Introduction

Chikungunya virus (CHIKV), an arbovirus transmitted by *Aedes* mosquitoes, can cause a clinical disease that resembles dengue and other arboviral infections [1]. Acute-phase signs and symptoms include fever, myalgia and rash, but severe arthralgia that may become chronic is the main hallmark of the disease [1]. An increase in Guillain-Barré Syndrome (GBS) was also observed during a CHIKV outbreak in French Polynesia, indicating that this arbovirus may be associated with severe neurological outcomes [2]. Clinical diagnosis is difficult, especially where co-circulation of other arbovirus such as dengue (DENV) and Zika (ZIKV) viruses occurs. In addition, as sequential arboviral infections and even co-infections could play a role in severe clinical manifestations, there is a need of a better understanding of multiple arboviruses transmission dynamics [3].

Brazil has been in the spotlight for arbovirus transmission, especially since epidemics of Zika virus (ZIKV) in early 2015 [4–6] were followed by outbreaks of GBS in adults and microcephaly in newborns [7,8]. During January-September 2016 (up to epidemiological week 37), Brazil recorded 200,465 ZIKV cases, 236,287 CHIKV cases and 1,438,624 DENV cases [9]. Oropouche and Mayaro virus infections have been identified sporadically in the country, restricted so far to the North and Midwest region [10–14], whereas Yellow Fever occurs endemically in the Amazon region with occasional transmission in the Midwest, South and Southeast regions, with 322 reported cases from July 2014 to June 2015 in Brazil [15]. Salvador, the largest city in the northeastern region of Brazil, and the capital of Bahia State, was one of the cities most affected by ZIKV. While there was widespread occurrence of ZIKV cases in Salvador, transmission of CHIKV appeared to have been much less intense, to the extent that an outbreak was not detected by local health authorities, as during the period an outbreak of acute exantematous illness (AEI) attributed to ZIKV occurred over 14,000 AEI cases were reported in contrast to 58 CHIKV cases reported in Salvador [6]. This is intriguing, since CHIKV has caused large outbreaks in most places where it is introduced [16–19], and CHIKV cases were first detected in Brazil in May 2014, in Feira de Santana, a city located approximately 100 km north of Salvador [20]. In Feira de Santana, CHIKV reached outbreak levels, with 4,088 reported cases in 2015 (an incidence of 668.0 cases/100,000 inhabitants) [21]. In contrast, in Salvador, 1,240 CHIKV cases were reported in 2015 (an incidence of 42.7 cases/100,000 inhabitants, more than an order of magnitude lower) [21].

However, CHIKV transmission in Salvador may have been masked by the ZIKV, GBS and microcephaly outbreaks, which prevailed in the media and got much of the attention of health professionals. Here we describe the results of an investigation aiming to determine the intensity of transmission of CHIKV in Salvador, Brazil, during the period of occurrence of ZIKV, GBS and microcephaly outbreaks.

## Methods

In collaboration with the Salvador Secretary of Health and the State’s Central Laboratory of Public Health (LACEN-BA), we retrospectively analyzed all the serum sample results from Salvador patients that were tested for CHIKV at LACEN-BA between November 4, 2014 and April 19, 2016. Other than research and private laboratories, LACEN-BA is the sole public health laboratory in the State of Bahia capable of performing arbovirus diagnosis. It receives samples from patients suspected of arboviral disease from public health units throughout the state. The decision as to which patients’ serum samples are collected and sent for CHIKV testing is made by the attending physician, and follows the Brazilian guidelines for CHIKV suspicion, which is defined by sudden onset of fever (>38.5°C) and arthralgia or intense arthritis in residents or visitors of endemic or epidemic areas [22].

Serum samples collected through the fifth day of onset of symptoms were tested by CHIKV IgM ELISA [23] or by CHIKV RT-PCR [24], while those collected more than 5 days after onset were only tested using CHIKV IgM ELISA. CHIKV IgM ELISA testing was performed during the whole study period, whereas CHIKV RT-PCR was performed for samples from November 2014 to December 2015. Additionally, during the AEI outbreak attributed to ZIKV [6], samples sent to LACEN-BA due to AEI symptoms were also tested for CHIKV. We considered a sample positive for CHIKV if it tested positive by either ELISA or RT-PCR.

We constructed an epidemiological curve by epidemiological week of the date of serum collection, plotting the absolute and relative frequency of CHIKV detection. The percentage of positive samples was smoothed using a 5-week moving average, wherein the count of events for a given week was averaged with the values in the 2 previous and 2 following weeks to reduce week-to-week variation.

This investigation was performed using de-identified secondary laboratory data obtained by routine activities of the Epidemiological Surveillance Office/Municipal Secretariat of Health from Salvador, Bahia, Brazil. The study was approved by the Salvador Secretariat of Health and the Oswaldo Cruz Foundation Ethics Committee.

## Results

Of 3,042 serum samples from Salvador patients received by LACEN-BA for CHIKV testing during the study period, 2,656 (87%) were tested only using ELISA, 49 (2%) were tested only by RT-PCR, 31 (1%) were tested by both methods, and for 306 (10%) no result was available (either due to an insufficient sample or because it has not yet been tested). Samples that were not tested by at least one method (306) were excluded from analysis, resulting in a final count of 2,736 analyzed serum samples.

**Fig 1:**
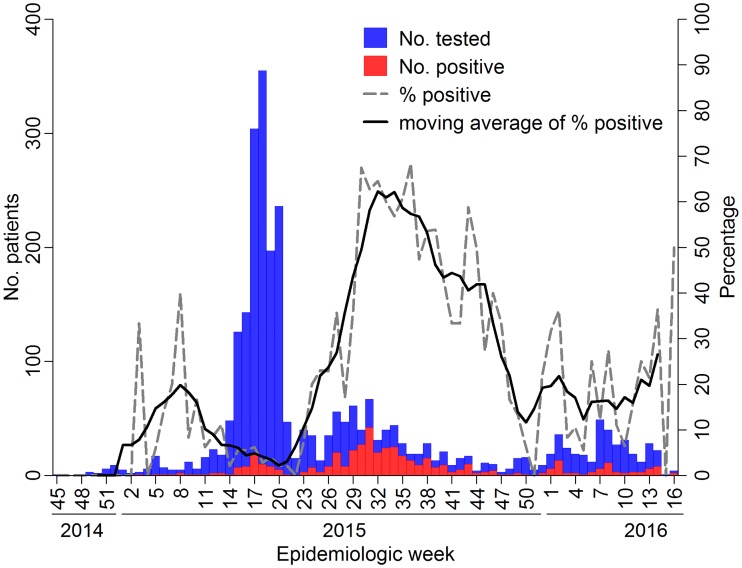
Epidemiological curve of absolute and relative frequencies of Chikungunya detection by epidemiological week, Salvador, Brazil.

In total, 456 (16.7%) of the 2,736 samples analyzed during the study period were positive for CHIKV. Of the samples tested by RT-PCR 45% (36/80) were positive and of those tested by ELISA, 15.7% (422/2,687) were positive. The first laboratory-confirmed chikungunya case detected through this study occurred in week 3 (January 23) of 2015, and the positivity rate increased steadily until week 8 (February 22–28) (Fig 1). This trend was then interrupted and reversed, coinciding with the vast increase in number of samples tested, reaching more than 350 samples during week 18 (May 3–9) of 2015. The positivity rate then increased again, starting in week 20 (May 17), and peaking during weeks 26–47 (June 28-November 28) of 2015, when more than 25% (up to 68% at week 36, September 6–12, 2015) of the tested samples were positive for CHIKV. The percentage of CHIKV positive samples from Salvador remained at levels of ~10–20% through the rest of 2015 and the first weeks of 2016.

Additionally, during the study period a total of 3,328 samples were tested for DENV (from December 2014 to April 2016) using IgM or NS1 ELISA (Panbio Diagnostics, Brisbane, Australia), of which 599 were positive. Of the 2,736 samples tested for CHIKV, 1,126 were also tested for DENV (from December/2014 to April/2016) and 158 (14.0%) were positive. Concomitant positivity for DENV and CHIKV was found for 37 samples (36 CHIKV positivity by ELISA and 1 by RT-PCR). A total of 129 samples were tested for ZIKV (from April/2015 to April/2016) by real-time RT-PCR [25], four of which were positive. Laboratory testing for both CHIKV and ZIKV was performed for 58 samples (collected between the end of July 2015 and April 2016), none of which were positive for ZIKV (three of the 58 were positive for CHIKV by IgM ELISA).

## Discussion

The increase in the frequency of CHIKV positive laboratory results among Salvador patients during 2015 suggest that the intensity of CHIKV transmission in the city followed the same temporal pattern observed for the laboratory exams, with CHIKV transmission likely peaking in August, shortly after the exanthematous illness outbreak attributed by excess to ZIKV only [6]. Although Salvador established a surveillance for CHIKV detection following Feira de Santana’s outbreak in 2014 [26], the virus’ introduction and subsequent spread in the city was not promptly noticed by the health authorities, because their main focus was on the AEI outbreak attributed to ZIKV, which affected about 14,000 people over a two month period [7]. Additionally, due to the overwhelming demand, especially during the AEI outbreak, laboratory testing was not performed in a timely manner. Therefore, the health authorities were only informed of the increase of CHIKV cases retrospectively.

The interruption of the ascending trend in CHIKV positivity in weeks 8–20 corresponds to the peak of the AEI outbreak attributed to ZIKV in Salvador (April 19–May 23, 2015) [7]. This probably accounts for the increase in the number of samples tested, given that suspected AEI cases were also tested for CHIKV. Despite the high number of samples tested, the frequency of CHIKV detection was relatively low (<10.0%), supporting the hypothesis that the AEI outbreak was associated mostly with ZIKV infections [6]. Thus, the low frequency of CHIKV detection during the AEI outbreak period should be interpreted with caution, since testing likely included primarily patients with AEI, rather than CHIKV suspected cases. Unfortunately, we cannot distinguish samples from this period that were sent to be tested because of a clinical suspicion of CHIKV from samples that were tested because of AEI. It is likely that the increase in CHIKV cases in Salvador in 2015 started before the first cases of AEI were detected, but the epidemiological curve lagged because testing targeted mostly AEI patients during this period. This possibility is supported by the fact that the first laboratory confirmed CHIKV cases were from January 2015.

This finding also suggests that the CHIKV transmission in Salvador was less explosive than the 2015 ZIKV outbreak (over 17,000 reported cases in nearly 10 weeks) (7), but, in contrast, was of longer duration and may have resulted in established endemic transmission, given that the percentage of CHIKV positive samples from Salvador remained at levels of ~10–20% through the rest of 2015 and the first weeks of 2016. It is also possible that the CHIKV outbreak reported here is under-estimated, while the ZIKV outbreak is over-estimated (i.e., all severe manifestations observed in Salvador were attributed almost entirely to ZIKV circulation). Even though the number of people infected by both viruses was certainly under-estimated given how surveillance of cases was assembled, health-seeking behavior and the general perception that AEI was a self-limited mild disease.

Salvador was one of the epicenters of ZIKV, GBS and microcephaly outbreaks in Brazil during 2015. A causal relation between ZIKV and the congenital disorders outbreaks has been established [27,28]. In French Polynesia, a case-control investigation also pointed to a link between prior ZIKV infection and GBS development [29], supporting a relation between the outbreaks of ZIKV and GBS in Brazil. However, CHIKV has also been previously related with GBS in both French Polynesia and Réunion Island [2,30]. Thus, our findings of intense CHIKV transmission in Salvador between June and November co-occurring with the period of the GBS outbreak in the city [7], support a possible connection between CHIKV infections and GBS development in Salvador.

Some limitations need to be acknowledged. First, the majority of the samples were tested only using IgM-based serology, and thus cross-reaction to other alphavirus has to be considered. However, although the occurrence of other alphavirus such as Mayaro have been described in the North and Midwest regions of Brazil [10,12], there is no evidence for their circulation in Salvador. Second, our epidemiological curve is based on the time when a sample was taken from the patient, which might not necessarily represent the time of infection, especially as CHIKV infections may result in chronic clinical manifestations and serum samples may have been collected for diagnosis a long time after disease onset. In this case, IgM antibodies would no longer be present and IgG-ELISA would be more appropriate. Also, although RT-PCR for acute-phase samples and IgM detection in paired samples would provide a more accurate diagnosis [31], a different algorithm for CHIKV testing was adopted due to limited resources. Third, this study included only cases that sought healthcare and whose attending physician requested laboratory testing for either CHIKV or differential diagnosis of an AEI, thus underestimating CHIKV cases. Additionally, with syndromic surveillance it is not possible to define accurately the etiology of cases, therefore laboratory testing is essential. In this study, we tried to address this limitation by analyzing the available laboratory results for all patients tested for CHIKV in Salvador, and our results are supported by previously published data showing that the AEI outbreak in Salvador that peaked in May was mainly due to ZIKV [6]. Yet, community-based studies using serological tests are needed to help better ascertain the intensity of CHIKV transmission, and there is an urgent need for ZIKV serological tests to accurately assess the intensity of ZIKV transmission. Fourth, CHIKV outbreaks in Feira de Santana appear to have occurred in two waves, the first in June-December 2014 and the second starting at January-2015 [32]. Since the first patients from Salvador to be tested for CHIKV infection were not tested until November 2014, we might have missed any earlier CHIKV transmission in Salvador during the first wave of transmission in Feira de Santana. Lastly, both viral isolation and genome sequencing are not routinely performed by LACEN-BA; thus, detailed information on strains responsible for this outbreak was not available. However, other studies have identified CHIKV infections in Bahia associated with the East-Central-South African (ECSA) strain [32,33].

Our findings reinforce the need for a better understanding of the co-circulation of these arboviruses. In such a setting, with high intensity of transmission of more than one arbovirus, co-infections may be common, as these viruses have the same vector, future studies are needed to better understand the role of sequential and co-infections in the severity of clinical manifestations. Failing of detect the co-circulation of other arbovirus in a timely fashion hampers the ability to implement actions to prevent and treat severe or chronic manifestations that may elapse, such as incapacitating chronic arthralgia in the case of CHIV infections, for example. In addition, the unrecognized co-circulation of other arboviruses could partially explain the occurrence of other severe outcomes in the region, such as GBS. Even with regard to microcephaly, Brazil authorities are now set to explore the country's peculiar distribution of Zika-related microcephaly [34]. The concentration of such cases in the Northeast, where all three arboviruses have been co-circulating needs to be considered (together with other risk factors). As co-circulation of arboviruses is likely occurring in several other tropical cities, researchers, physicians, and public health professionals must consider CHIKV as a differential diagnosis together with DENV and ZIKV when studying arbovirus transmission and disease, while examining suspected case patients and when performing surveillance in Brazil and elsewhere.

Clinical differential diagnosis between these arboviruses is difficult, as observed by Salvador’s experience during the AEI outbreak when ZIKV, DENV and CHIKV were co-circulating [6], are three capable of causing AEI. In such a scenario, syndromic surveillance can provide a rough estimate of disease transmission, but a syndromic laboratory assessment approach testing all patients with non-specific arboviral disease symptoms for DENV, ZIKV and CHIKV (such as multiplex testing), should be considered, if possible.

## Supporting Information

S1 Checklist STROBE checklist. (DOC)

S1 Table (XLSX)
